# Colchicine trial in PFAPA Syndrome and MEFV-negative patients

**DOI:** 10.1186/1546-0096-13-S1-O5

**Published:** 2015-09-28

**Authors:** C Kadhim, F Maiolini, L Cerrito, LL Sicignano, M Giovinale, E Verrecchia, F Gurrieri, M Genuardi, R Manna

**Affiliations:** 1 Catholic University of the Sacred Heart, Internal Medicine, Rome, Italy; 2 Catholic University of the Sacred Heart, Human Genetic, Rome, Italy

## Introduction

PFAPA Syndrome (Periodic Fever, Aphthous stomatitis, Pharingitis, and cervical Adenitis) is the most common periodic fever in childhood; the diagnosis is based on clinical criteria. Familiar Mediterranean Fever (FMF) is a monogenic autosomal recessive autoinflammatory disease, whose diagnosis is based on clinical elements, supported by MEFV genetic mutations. When there is only a mutation or no one, the patient undergoes a trial with colchicine for 4-6 months, and diagnosis is confirmed in case of clinical response and fever early recurrence after suspension. Current treatment of PFAPA is symptomatic. Febrile episodes show a rapid response to the administration of one or two doses of prednisone (1-2 mg/kg) or betamethasone (0.1-0.2 mg/kg). Total requirement of steroid increases over time, and the frequency of attacks worsens the quality of life of patients. In literature, the prophylaxis of PFAPA febrile attacks with colchicine (0.5-1 mg/day) has been tested only on a few patients, with controversial results.

## Objectives

Considering the similarities between FMF MEFV-negative patients (MEFVneg) and PFAPA patients, we aimed to demonstrate that colchicine is effective in PFAPA too: positive response was evaluated in terms of reduction in frequency >50% and severity of attacks >50%.

## Materials and methods

We conducted a prospective cohort study (from September 2012, still ongoing), comparing two groups: 67 MEFVneg and 51 PFAPA patients. 36 of the latter group underwent colchicine trial, after obtaining informed consent.

## Results

We assessed the response of PFAPA patients to colchicine preventive treatment: good response was observed in 75% (27 patients of 36), and a non-response in 25% (9 pts). The effective treatment rate of MEFVneg is 100%, by definition. The average dose of colchicine administered in PFAPA was 1.14 mg/day, compared to MEFVneg (1.34 mg/day). The dose per kilogram of body weight is 0.020 mg/kg/day in both groups. We can state that the colchicine dose requirement in PFAPA coincides to the one of FMF patients.

## Conclusion

Our study showed that colchicine regimen is effective in 75% of cases. Prophylaxis with colchicine should be offered to all PFAPA patients, instead of steroids or other symptomatic therapy (as paracetamol or ibuprofen), before the treatment with anti-IL1β biologic drugs, with considerable savings in pharmacoeconomics.

